# Hybrid confocal fluorescence and photoacoustic microscopy for the label-free investigation of melanin accumulation in fish scales

**DOI:** 10.1038/s41598-022-11262-0

**Published:** 2022-05-03

**Authors:** George J. Tserevelakis, Michalis Pavlidis, Athanasios Samaras, Georgios D. Barmparis, Kostas G. Mavrakis, Ioannis Draganidis, Athanasios Oikonomou, Eleftheria Fanouraki, Giorgos P. Tsironis, Giannis Zacharakis

**Affiliations:** 1 Institute of Electronic Structure and Laser, Foundation for Research and Technology Hellas, N. Plastira 100, 70013 Heraklion, Crete, Greece; 2 Department of Biology, University of Crete, Voutes University Campus, 70013 Heraklion, Crete, Greece; 3 Institute of Theoretical and Computational Physics and Department of Physics, University of Crete, P.O. Box 2208, 71003 Heraklion, Greece

**Keywords:** Optical techniques, Imaging and sensing, Microscopy, Ichthyology

## Abstract

Lower vertebrates, including fish, can rapidly alter skin lightness through changes in melanin concentration and melanosomes’ mobility according to various factors, which include background color, light intensity, ambient temperature, social context, husbandry practices and acute or chronic stressful stimuli. Within this framework, the determination of skin chromaticity parameters in fish species is estimated either in specific areas using colorimeters or at the whole animal level using image processing and analysis software. Nevertheless, the accurate quantification of melanin content or melanophore coverage in fish skin is quite challenging as a result of the laborious chemical analysis and the typical application of simple optical imaging methods, requiring also to euthanize the fish in order to obtain large skin samples for relevant investigations. Here we present the application of a novel hybrid confocal fluorescence and photoacoustic microscopy prototype for the label-free imaging and quantification of melanin in fish scales samples with high spatial resolution, sensitivity and detection specificity. The hybrid images are automatically processed through optimized algorithms, aiming at the accurate and rapid extraction of various melanin accumulation indices in large datasets (i.e., total melanin content, melanophores’ area, density and coverage) corresponding to different fish species and groups. Furthermore, convolutional neural network-based algorithms have been trained using the recorded data towards the classification of different scales’ samples with high accuracy. In this context, we demonstrate that the proposed methodology may increase substantially the precision, as well as, simplify and expedite the relevant procedures for the quantification of melanin content in marine organisms.

## Introduction

Coloration patterns and skin morphological and physiological changes play a crucial role in the biology, physiology, behavior and ecology of fish^1,2^. Lightness (brightness), hue and chroma sophisticated properties of fish skin are based on the presence, density, distribution and mobility of pigment cells (chromatophores) and the concentration of associated chemical compounds (pigments). Chromatophores can be classified into two main categories: (1) the light-absorbing, melanophores, erythrophores, xanthophores and cyanophores, and (2) the light-reflecting, leucophores and iridophores^3^. Melanophores, in particular, consists of relatively large melanosomes that contain the light-absorbing pigment melanin, producing dark skin colors. Lower vertebrates, including fish, can rapidly alter skin lightness depending on various factors, including background color, light intensity, ambient temperature, social context and husbandry practices^1,4^. Such rapid changes are due to changes in the mobility (aggregation or dispersion) of the melanosomes within the melanophore and are termed physiological color changes^1,3–6^.

In aquaculture conditions, skin coloration, and especially lightness, has been related to product quality, animal welfare^2,7^, as well as to consumer’s perception and economic value of marketable-sized fish^8–11^. Changes in skin lightness are closely related to the melanin content and the number and shape of melanophores^3^. The number of melanophores is considered to be more or less stable, with only slight changes after exposure to environmental factors^3^. On the contrary, the area of skin covered by the melanosomes, i.e., whether melanin is aggregated or dispersed within the cell, is highly variable^1,3,4^.

Changes in melanin concentration and melanosomes’ mobility are also observed following exposure of fish to environmental, social and husbandry (in the case of reared fish) acute or chronic stressful stimuli. Furthermore, are related to health status, since intra-specifically divergent melanin-based color morphs have been related to differential exposure to parasites, and, once infected, to differential abilities to cope with the infection^12,13^. Moreover, a relationship between melanin-based color morphs and stress response has been described in rainbow trout^14^ and Atlantic salmon^12^, while genetic correlations between skin pigmentation and growth have also been documented in rainbow trout^15^. Finally, due to the fact that individuals with higher melanin concentration and/or dispersion have more protection against UV-radiation than conspecifics with lower melanin, dark individuals are considered to be less affected from global warming^16^. This difference could potentially affect population dynamics in an evolutionary or plastic manner.

Although quantification of skin chromaticity parameters in fish species can be accurately estimated either in specific fish areas using colorimeters^2^ or at the whole animal level using image processing and analysis software^17^, measuring the melanin content or melanophore number and coverage in fish skin has been proven challenging until now. Melanin is insoluble in water and shows an irregular structure creating an obstacle for chemical analysis, particularly quantification^8^. Additionally, in order to quantify melanin, fish need to be euthanized to obtain skin samples. Then melanin has to be extracted, determined indirectly (against a sepia melanin synthetic standard) and expressed roughly as melanin content per area of skin^4,5,18–20^. Another, more accurate, methodology is based on the chemical degradation of eumelanin to pyrrole-2,3,5-tricarboxylic acid (PTCA) and of pheomelanin to aminohydroxyphenylalanine isomers (AHP), which can be quantified by High Performance Liquid Chromatography^21,22^. Melanophores number and melanosomes coverage on fish scales, on the other hand, is traditionally measured optically, through stereoscopical imaging of the scales and image processing and analysis software^5,19,20,23^, which is a challenging, laborious, and relatively slow process. In this context, novel techniques for fast and accurate analysis of melanin content and melanophores number in fish scales could significantly contribute to color analysis in fish.

Biomedical photoacoustic (PA) microscopy is a powerful and rapidly expanding imaging modality which exploits the weak attenuation of ultrasonic waves to provide imaging contrast deep inside tissues^24^ with 100% relative sensitivity to optical absorption^25^. In this direction, the capabilities of PA microscopy have been recently demonstrated in several applications involving the in-vivo acquisition of valuable anatomical, molecular, functional, and flow dynamic information^26,27^, towards the elucidation of fundamental mechanisms such as cancer formation and growth^28,29^, the high-resolution monitoring of drug delivery processes^30^, as well as, the detailed imaging of ocular structures through specialized PA ophthalmoscopes^31,32^.

The underlying physical mechanism behind this diagnostic technique is the PA effect, which can be simply described as the formation of acoustic waves following the absorption of light with temporally-modulated intensity. More specifically, the irradiation of biological specimens through laser pulses with a typical width of a few nanoseconds, induces a local temperature rise of several milliKelvin in highly absorbing regions^33^. Under such excitation settings, the transient response of the medium has the form of a rapid thermoelastic expansion, which in turn, generates an initial pressure perturbation propagating as broadband acoustic waves with predominant frequency content in the MHz regime. These ultrasonic waves are usually recorded by employing the same detection equipment (e.g., piezoelectric elements) as in traditional ultrasound imaging^34^. When thermal and stress confinement conditions are sufficiently met (i.e., no considerable loss of heat and pressure is observed in the excitation region throughout the optical pulse duration), the peak-to-peak amplitude of the PA waves is directly proportional to the absorption coefficient of the medium for the excitation wavelength^35^. The respective PA microscopy images are formed without using any reconstruction algorithms such as back-projection, either by restricting the excitation area through the well-defined optical focus of the laser beam (optical-resolution mode), or by constraining the signal detection region following the integration of spherically focused ultrasonic transducers (acoustic-resolution mode). These two PA microscopy approaches can be employed according to the required trade-off between the imaging detail and the maximum imaging depth, as optical-resolution mode may reveal fine spatial features up to 300 nm within depths not exceeding 1 mm, whereas the acoustic-resolution mode allows for deeper investigations (~ 3 mm) at the cost of a significantly reduced resolving power (~ 25–30 μm) in the lateral dimensions^26^.

The most prominent biological absorbers which emit strong PA signals following their excitation with visible wavelengths are melanin and hemoglobin^36^, providing thus rich intrinsic contrast of pigmented layers or tumors and capillary vessels respectively. As regards melanin, several previous studies have demonstrated the promising capabilities of PA microscopy on the detection, monitoring and quantification of melanin depositions in skin^37^, eye^38^, circulating melanoma cells^39^ and several animal models including mice and zebrafish^40–42^. With the exception of hemoglobin, all of the typical endogenous tissue chromophores present virtually negligible absorption coefficients in the visible part of the optical spectrum when compared to melanin. For a usual PA excitation wavelength at 532 nm, melanin is characterized by an absorption coefficient of approximately 500 cm^−1^^43^, which is at least 3 orders higher than the respective values for lipids (~ 0.01 cm^−1^)^44^, collagen (~ 0.25 cm^−1^)^45^ and elastin (~ 0.5 cm^−1^)^46^. Within this context, the high potential of PA microscopy concerning the sensitive detection of endogenous optical absorbers in tissues, has been effectively combined with several pure optical techniques in multimodal instruments, such as confocal fluorescence microscopy^47–49^, non-linear imaging^50–52^ and optical coherence tomography (OCT)^53–56^. These novel hybrid approaches have allowed for the extraction of complementary information in several types of biological specimens, including ocular and vegetative tissues, aiming at an augmented PA diagnosis with high applicability in various biomedical studies.

In this work, we present the application of a hybrid optical-resolution PA and confocal fluorescence (CF) microscopy system for the label-free imaging and quantification of melanin content in fish scales with high spatial resolution, sensitivity and detection specificity. We initially demonstrate that the PA signals are predominantly generated by the melanophores of the scale, and subsequently show that the detected PA amplitude is directly proportional to the local melanin concentration in specially designed phantom samples simulating fish scales. The recorded hybrid images are automatically processed using novel optimized algorithms, aiming at the accurate and rapid extraction of various melanin accumulation indices in large datasets corresponding to different fish species and groups. Furthermore, convolutional neural network-based algorithms are employed for the classification of these scales with high accuracy. Within this framework, we provide evidence that the proposed imaging methodology has the potential to increase substantially the accuracy, as well as, simplify and expedite the relevant procedures for the quantification of melanin in marine organisms.

## Materials and methods

### Scale sampling and storage

Scales from three teleost fish species were collected to develop a novel hybrid CF and PA microscopy platform in quantifying the content of melanin and the number of melanophores in fish scales. The species from which scales were collected were farmed European sea bass (*Dicentrarchus labrax*) of mean weight (± SD) of 135.8 g (± 18.7), farmed gilthead seabream (*Sparus aurata*) of mean weight (± SD) of 202.5 f. (± 26.4), and wild-caught common pandora (*Pagellus erythrinus*) of mean weight (± SD) of 190.8 g (± 26.2). Species were chosen based on (a) their differences in skin lightness (high, intermediate and low), (b) high commercial value and (c) availability in the market.

For this purpose, scales (n = 10 to 20 per individual) were collected from the dorsal area, above the lateral line of fish using a forceps. Scales were fixed in 4% buffered formaldehyde and stored at 70% ethanol as previously described^57,58^. In total, 100 scales from each species were collected and analyzed for melanin content under a hybrid CF and PA microscopy setup. Moreover, a second experiment was conducted on porgies purchased from two different fish suppliers from Crete (thereinafter termed as S1 and S2) based on macroscopic differences in skin lightness. Scales were collected as previously described and were analyzed under the hybrid CF and PA microscopy setup. The conducted research did not involve any experimentation in live animals. All tests were performed in specimens purchased by local super-markets or fish markets.

### Hybrid CF and PA microscopy setup

**Figure 1:**
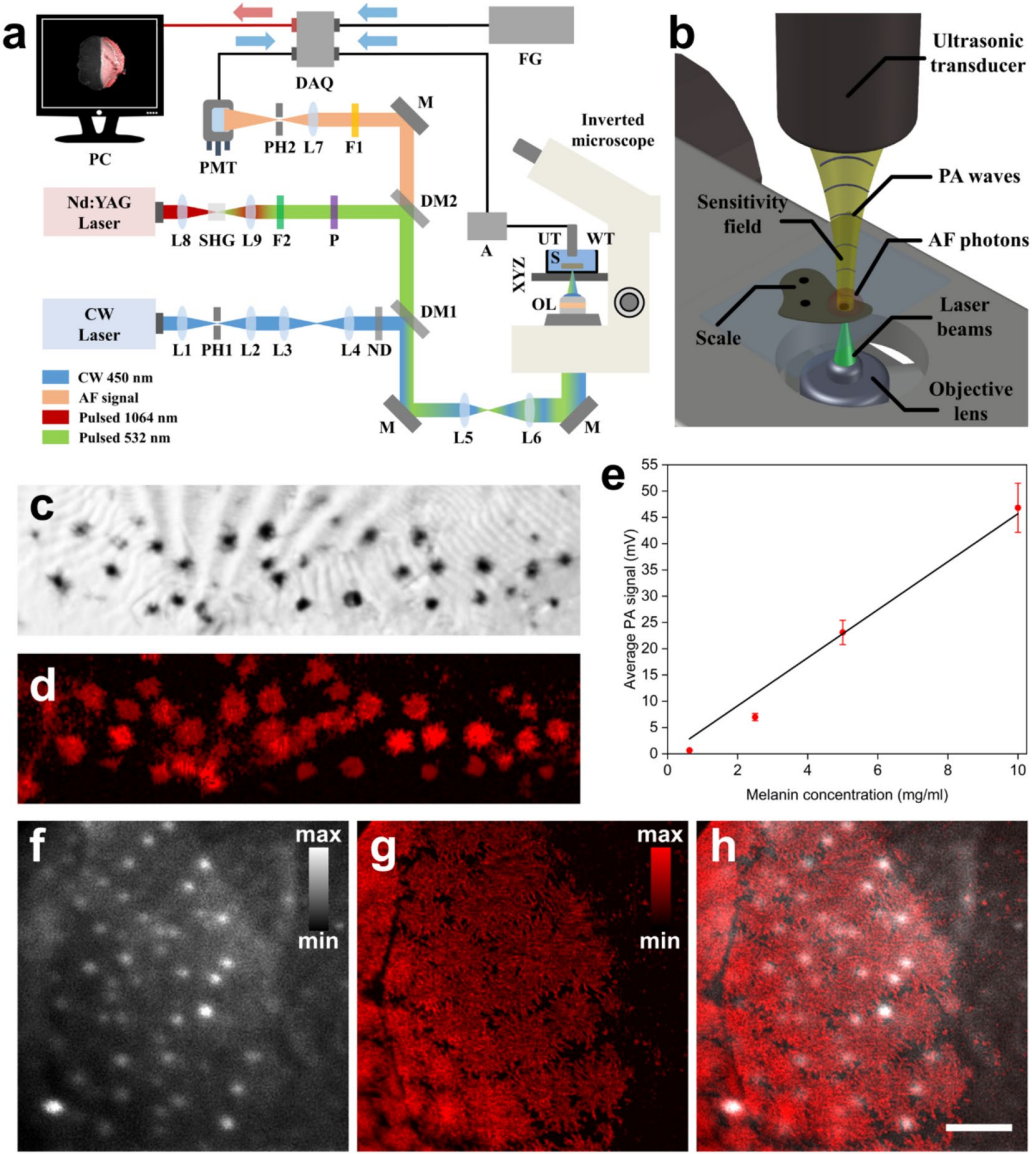
Evaluation of hybrid microscope’s imaging performance on the investigation of fish scales. (**a**) Schematic representation of the developed hybrid microscopy system. *L (1–9)* lenses, *M* mirror, *PH (1–2)* pinholes, *ND* neutral density filters, *DM (1–2)* dichroic mirrors, *F (1–2)* optical filters, *PMT* photomultiplier tube, *SHG* second harmonic generation crystal, *P* linear polarizer, *OL* objective lens, *XYZ* 3D translation stages, *WT* water tank, *S* scale sample, *UT* ultrasonic transducer, *A* amplifier, *DAQ* data acquisition card, *FG* function generator, *PC* recording computer. (**b**) A close-up illustration of hybrid imaging procedure during the scanning of a fish scale sample. (**c**) Brightfield image of the posterior field in a porgy scale (field of view: 1.71 by 0.44 mm^2^). (**d**) MAP PA reconstruction of the same region as in **c** using a pixel size of 6 μm. (**e**) Plot of average PA amplitude versus melanin’s concentration in agarose phantom samples. Error bars indicate a ± 5% signal deviation due to pulse energy variability. Solid black line represents a linear fitting of the data points (R^2^ = 0.99). (**f**) High-resolution AF image of the posterior field in a sea bass scale. (**g**) Respective PA image of the same region. (**h**) Hybrid image integrating CF (grayscale) and PA (red color) contrast modes. Scale bar is equal to 200 μm.

The hybrid microscopy setup (Fig. 1a) integrates two excitation beams and two corresponding optical paths, each of them dedicated to CF and PA microscopy modalities respectively. The paths are merged using suitable dichroic mirrors (DM1 and DM2) exactly before the microscopy platform, to achieve the simultaneous excitation and detection of AF and PA signals, which in turn provides dual contrast images. CF microscopy modality employs a compact continuous wave (CW) diode-pumped laser module (CPS450, Thorlabs, Newton, NJ, USA; output power 4.5 mW) emitting at 450 nm, as AF excitation source. Due to its highly elliptical shape, optical radiation is spatially filtered using a telescope consisted of two positive lenses (L1 and L2; F1 = 50 mm, F2 = 40 mm) and a 25 μm diameter pinhole (PH1), to obtain a near-Gaussian beam profile. An optical system integrating another two positive lenses (L3 and L4; F3 = 75 mm, F4 = 50 mm) at a variable relative distance is subsequently used to control the divergence of the beam, adjusting precisely the axial overlap of optical foci on sample’s plane. The spatially filtered radiation is attenuated after passing through a set of neutral density filters (NDK01, Thorlabs, Newton, NJ, USA), to avoid any photodamage effects on the examined specimen. The beam is then reflected by a long-pass dichroic mirror (DM1; DMLP505, Thorlabs, Newton, NJ, USA; cut-off wavelength: 505 nm) and guided into a properly modified inverted optical microscope (Labovert, Leitz, Wetzlar, Germany), following a 3X expansion by a telescope (L5 and L6; F5 = 50 mm, F6 = 150 mm). An objective lens (OL; Achromat 8X, LOMO, St. Petersburg, Russia; air immersion; NA: 0.2) focuses the light directly on the scale (S), which is placed at the bottom of an optically transparent water tank (WT) and firmly fixed using a thin layer of ultrasound gel. The tank is further attached on a high precision 3D translational motion system (XYZ) composed of a fast motorized XY stage (8MTF-75LS05, Standa, Vilinius, Lithuania) raster-scanning the sample over the beam focus, and the built-in manual Z-control of the microscope, through which, the imaging focal plane is selected. A part of the back-scattered AF radiation is collected by the objective lens and transmitted through the dichroic mirrors DM1 and DM2 (DMLP550, Thorlabs, Newton, NJ, USA; cut-off wavelength: 550 nm), as well as, a long pass filter (F1; FGL570, Thorlabs, Newton, NJ, USA; cut-off wavelength: 570 nm) to cut-off the reflected excitation light. Using a positive lens (L7; F7 = 30 mm), AF radiation is then focused through a 50 μm diameter pinhole (PH2), to effectively reject any out of focus signals. The remaining in-focus AF light is finally detected by a low-noise photomultiplier (PMT) module (H6780-20, Hamamatsu, Hamamatsu City, Japan). The generated signals are recorded by a high-speed data acquisition (DAQ) card (PCIe-9852, ADLINK, Taipei, Taiwan; sampling rate: 200 MS/s; bandwidth: 90 MHz) and subsequently stored in a computer, following the averaging of 20 measurements for signal to noise ratio (SNR) improvement.

As regards the PA imaging path, a variable repetition rate pulsed Nd:YAG laser emitting at 1064 nm (QIR-1064-200-S, CrystaLaser LC, Reno, NV, USA; maximum pulse energy: 29.4 µJ, pulse duration: 10 ns) has been employed as an excitation source. The laser is externally triggered by an arbitrary function generator (FG; 33600A Series Trueform, Keysight Technologies, Santa Rosa, CA, USA) at a selected pulse repetition rate of 5 kHz, synchronizing also data acquisition and raster scanning procedures. The beam is focused on a LBO (Lithium triborate) Second Harmonic Generation (SHG) crystal (Castech Inc, Fuzhou, China) to be partially transformed into a 532 nm wavelength, and subsequently collimated by a second lens (L8 and L9; F8 = F9 = 100 mm). A band-pass filter (F2; FF01-531/40-25, Semrock, Rochester, NY, USA) is used to reject the remaining fundamental near infrared radiation, whereas the transmitted visible wavelength is sufficiently attenuated by passing through a linear polarizer (P; LPVISE200-A, Thorlabs, Newton, NJ, USA). Subsequently, the PA excitation beam is reflected by DM2 but transmitted through DM1 to overlap spatially with AF excitation light. After passing through the beam expansion telescope (L5 and L6), pulsed radiation is finally focused by the same objective lens (OL) on the sample under observation. The generated PA waves are detected by a 20 MHz central frequency spherically focused piezoelectric ultrasonic transducer (UT; V373-SU, Olympus, Tokyo, Japan; − 6 dB bandwidth: 13.3–32.9 MHz, Focal distance: 31.3 mm), which is immersed into the water tank in a confocal and coaxial configuration with respect to the optical focus. The distilled water in the tank serves as a coupling medium between signal source and the detector, ensuring the effective transmission of PA waves. The generated signals are enhanced by two low-noise RF amplifiers (A; TB-414-8A+, Mini-Circuits, Camberley, England; gain: 31 dB) connected in series to provide a total gain of 62 dB, prior their digitization and recording using a second channel of the DAQ card. To enhance the SNR level, 20 waveforms are averaged for the acquisition of each pixel value comprising the maximum amplitude projection (MAP) PA image. The total time required for the recording of a typical 400 by 400 pixels hybrid CF and PA image is approximately 15 min. Pulse energy on sample’s focal plane is measured at 120 nJ, whereas the respective optical power of the CW laser at 450 nm is approximately equal to 400 μW. The lateral resolution of the system is ultimately determined by the optical diffraction limit, and is estimated at around 2.5 μm, (PA modality) and 2.2 μm (CF modality), taking into account the underfilling of the objective lens which yields an effective NA of 0.1. In the same respect, the depth of focus for the PA modality can be calculated at ~ 50 μm, which is considered adequate enough for the homogeneous detection of melanophores on the surface of the scale. Finally, a typical laser fluence over the focal plane can be estimated by dividing the pulse energy by the surface of the laser spot (~ 20 μm^2^), which corresponds to a value equal to 0.6 J/cm^2^. Control and synchronization of the hybrid microscope has been performed using custom-developed programs. Finally, all recorded data have been processed by means of MATLAB environment and ImageJ open-source Java-based software.

### K-means clustering algorithm

A custom-made k-means clustering algorithm has been developed in MATLAB programming environment aiming at the automated detection of fish scale area in CF microscopy images. The selected region is further used for the rapid and accurate quantification of PA data, allowing for the extraction of melanin accumulation indices and the statistical processing of a large number of specimens. K-means is an iterative algorithm that tries to partition a dataset into k pre-defined distinct non-overlapping subgroups where each data point belongs to only one group. An analytical description of the k-means algorithm can be found elsewhere^59^. However, a short explanation of the clustering process can be summarized in the following steps: (a) Specification of cluster number k. (b) Assignment of some initial centroid values (arithmetic mean of data points) for each cluster either randomly or by providing predetermined values. (c) Estimation of the sum of the squared distance between data points and all centroids. (d) Assignment of each data point to the nearest cluster (centroid). (e) Computation of the centroids for the clusters by taking the average of data points that belong to the cluster. (f) Iteration of steps c, d and e until there is no change to the estimated centroids.

A pre-processing of the normalized to the maximum CF images is initially performed by applying a 2-D median filter (medfilt2; 10 by 10 neighborhood around each pixel) and a non-linear gamma-correction operation (γ = 0.2) to suppress the high spatial frequencies and smooth the varying brightness values across the scale. The CF image matrix is subsequently reshaped into a row vector prior the application of k-means intrinsic MATLAB operation, which attempts to partition data points into two clusters (i.e., scale area and background) using initial centroid values equal to 0.7 and 0.5 respectively. The average of the estimated centroids for the two clusters is then used as a brightness threshold to generate a 2-D binary mask marking the scale area. The mask is further corrected for possible “hole” artifacts using the intrinsic “imfill” MATLAB command and the “noholes” option. As more than a single region can appear in the mask due to background noise, the algorithm detects and chooses exclusively the largest area representing the fish scale with high accuracy in all CF images. The corresponding PA data falling into the selected scale area are finally quantified to generate different melanin accumulation indices which are representative of total melanin content, total melanophore area, density and % coverage, as well as, the average melanin content per point.

### Machine learning model for image classification

A Convolutional Neural Network (CNN) was trained to classify a dataset of 300, 400 by 400 pixels, single-channel, PA images (100 images of each fish scale class, i.e., common pandora, sea bass, and gilthead seabream). Initially, we randomly split the original dataset into a training plus validation (80%) and a test (20%) set. Then, we separate the training and the validation set by randomly splitting it into a training set (90%) and a validation set (10%). This process concludes with a training set of 216 images, a validation set of 24 images, and a test set of 60 images. To increase the number of images in the training set, a data augmentation method was applied by flipping the original images once horizontally (left–right) and once vertically (up–down), resulting in a set of 648 training images. Further augmentation (firstly horizontally and then vertically on the output image) was also checked but without improving our results.

Several different architectures and combinations of the hyper-parameters of models were tested to conclude to the one with the best performance. That model takes as input a single-channel 400 by 400 PA image and passes it through a feature extraction network that consists of six convolutional layers containing 4, 8, 16, 32, 64 and 128, 3 by 3 pixels, kernels respectively. A 2 by 2 pixels stride is applied on the first convolutional layer and a 3 by 3 max pooling layer after the second convolutional layer, to further reduce the size of the input image. Each one of the other convolutional layers is followed by a max pooling layer with a 2 by 2 kernel. The output of the feature extractor is then flattened and connected to a fully connected classifier containing two layers of 100 and 50 nodes respectively, leading to a 3 nodes output layer with a soft-max activation function.

The model was created using Keras^60^ as implemented in TensorFlow 2.4^61^ and compiled with an Adam optimizer^62^ with a learning rate of 0.001 and a categorical cross entropy loss function. The training process of the model was monitored on the validation set and an early stopping criterion was applied on the training loop with a patient of 20 epochs to avoid overfitting.

## Results

### Hybrid CF and PA microscopy platform

The hybrid CF and PA microscopy system presented in this study has been developed by modifying a conventional inverted optical microscope (Fig. 1a, “Materials and methods” section). CF imaging mode employs a CW diode laser module emitting at 450 nm for the efficient excitation of the investigated scale samples. Initially, the beam is spatially filtered, attenuated and reflected into the microscope by a dichroic mirror following its expansion by a telescope system consisting of two positive lenses. Optical radiation is subsequently focused on the sample by an objective lens to excite AF photons, part of which are collected back by the lens and pass through the dichroic mirrors of the system. Finally, the AF light is spatially filtered using a pinhole to reject the out of focus signals blurring the image, before being detected by a sensitive photomultiplier tube (PMT). On the other hand, PA imaging mode employs a Q-switched Nd:YAG laser emitting nanosecond pulses at 1064 nm wavelength, which is partially converted into 532 nm using a Second Harmonic Generation (SHG) crystal. The generated radiation is subsequently filtered, attenuated and guided towards the microscope through dichroic mirrors to overlap spatially with the CW beam. Following its expansion by the telescope, pulsed beam is focused by the objective lens on the sample which is in turn placed at the bottom of an optically transparent water tank. The generated PA signals are detected by a spherically focused immersion ultrasonic transducer in a confocal and coaxial configuration in respect to the optical focus (Fig. 1b). Hybrid images are generated by raster scanning the specimen over the optical foci of the two beams using a motorized XY stage, achieving a point-by-point acquisition over the region of interest.

### Evaluation of hybrid microscope performance

We have initially investigated the relative imaging performance of the developed hybrid microscopy system in terms of contrast specificity, dependence of PA amplitude on local concentration of melanin, as well as, the discrimination of fine spatial features in fish scale samples. A brightfield image showing the posterior field of a porgy scale is presented in Fig. 1c, covering a total region of 1.71 by 0.44 mm^2^. Dispersed skin melanophores are visible as individual spots of various sizes, or in aggregate form especially at the central part of the image. Figure 1d demonstrates a MAP PA reconstruction of the same area using a pixel size equal to 6 μm, highlighting the direct correspondence with Fig. 1c as regards the accurate detection of melanophores in the examined sample. It is apparent that melanin provides the predominant imaging contrast of PA modality among other chromatophores, as a result of its high optical absorption coefficient in the visible range. Having qualitatively identified the main signal sources in fish scales, we proceeded to the investigation of PA amplitude dependence on local melanin concentration. In this direction, we imaged four agarose-based phantom samples containing melanin at different relative concentrations ranging from 0.625 to 10 mg ml^−1^ using a pixel size equal to 20 μm (see “Materials and methods” section). A representative statistical sample of 2500 pixels was selected in each recorded MAP image for the estimation of an average peak-to-peak PA amplitude corresponding to each sample. Figure 1e shows a plot of the average PA signal versus melanin concentration in the agarose phantoms. Error bars indicate a typical ± 5% signal deviation due to the pulse energy variability of the laser excitation source. Data points have been fitted by a linear function (solid black line; slope: 4.57 mV per mg ml^−1^; R^2^ = 0.99) to highlight the direct proportionality of recorded PA amplitude with the local concentration of melanin. Finally, we have recorded high-resolution hybrid images of the posterior field in a sea bass scale sample, demonstrating the capabilities of the developed microscopy system on the extraction of fine spatial details. A field of view of 1 by 1 mm^2^ was selected and sampled using 400 by 400 pixels (pixel size: 2.5 μm). Figure 1f depicts an AF image of the scanned area, presenting several dispersed bright spots with a diameter of ~ 20 μm over a rather homogeneous signal background. These high-emission regions most probably correspond to known autofluorescent chromatophores such as xanthophores, containing pteridines and carotenoids of high quantum yield. On the other hand, the lower intensity AF background can be attributed mainly to collagen, which is found at high concentrations in fish scales. Figure 1g shows the respective PA image for the same region, providing the visualization of densely packed melanophores with high sensitivity and contrast. The spatial resolution of the image is sufficient enough to distinguish the dispersion of melanosomes to the periphery of the cells, as well as, local inhomogeneities of melanin’s distribution within melanophores. A hybrid microscopy image incorporating both AF and PA modalities is finally presented in Fig. 1h, to provide multiparametric information of the examined fish scale with high contrast complementarity in an absolutely label-free diagnostic approach.

### Automated scale selection using k-means clustering

**Figure 2:**
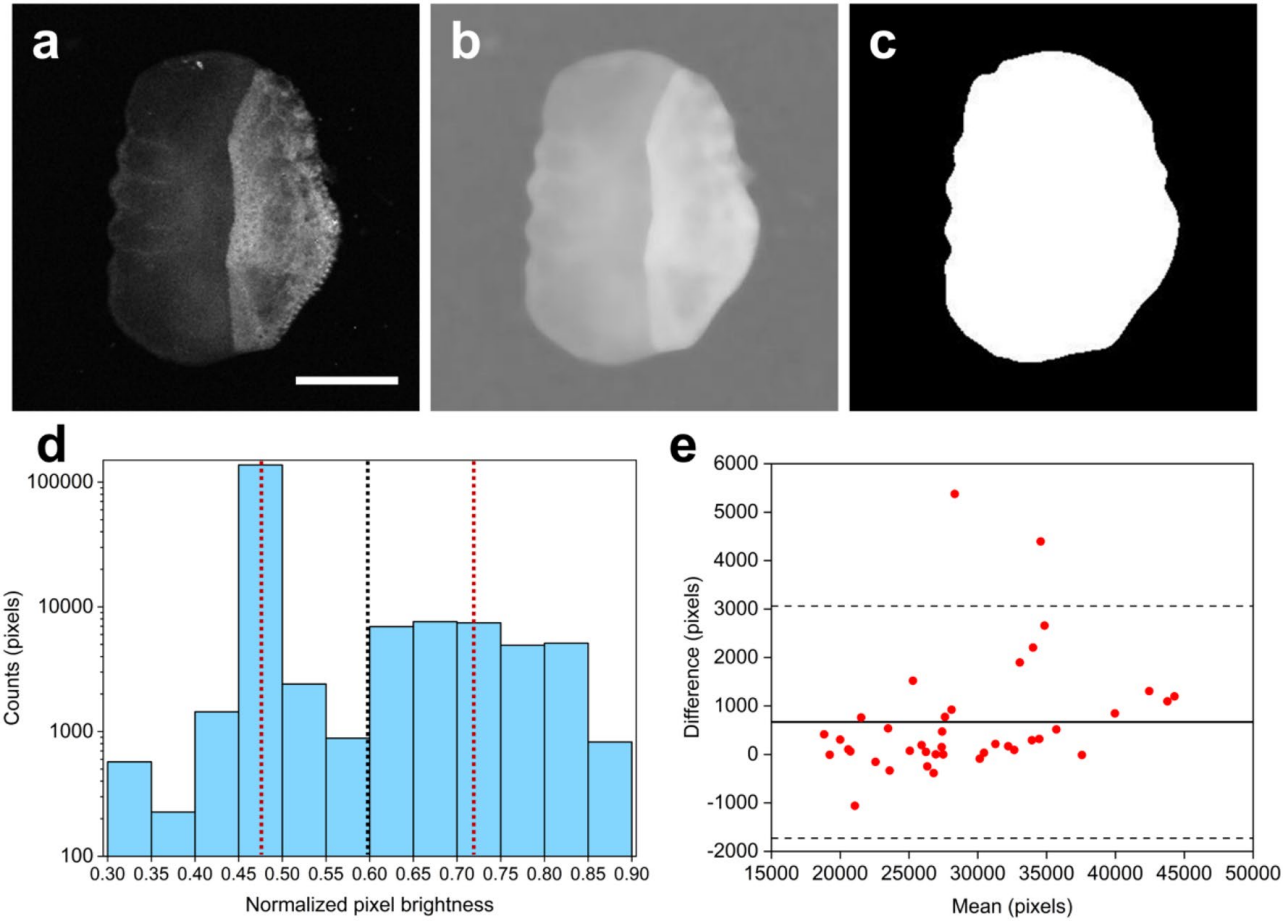
K-means based image processing algorithm for the automated selection of scale area. (**a**) Original CF image of a seabream scale. Scalebar is equal to 2 mm. (**b**) Pre-processed image of the scale shown in (**a**) by applying a 2-D median filter and a gamma-correction operation. (**c**) Respective binary mask generated by the k-means clustering algorithm, marking the scale area. (**d**) Histogram of normalized pixel brightness values for the pre-processed image shown in (**b**). Red dashed vertical lines show the final centroid predictions for the scale area (0.718) and the background (0.472) respectively. Black dashed vertical line stands for the average value (0.595) of the two centroids serving as a brightness threshold for the differentiation of the scale region. (**e**) Bland–Altmann plot for the evaluation of the agreement between manual selection of the scale area and automated k-means algorithm’s prediction. Solid black line corresponds to the mean value of differences (667 pixels), whereas dashed lines indicate the 95% confidence intervals (− 1728 and 3062 pixels respectively).

An automated image processing algorithm based on standard k-means clustering method has been implemented for the quantification of AF and PA data and the extraction of melanin accumulation indices in fish scales (see “Materials and methods” section). The algorithm takes advantage of the AF signal distribution in CF images to detect the scale with high accuracy, measuring also the corresponding PA signals in the selected area. Figure 2a shows a CF image of a seabream scale characterized by an inhomogeneous spatial distribution of AF signals, with the posterior field presenting an increased emission intensity most probably due to the higher collagen concentration in this region. The image is initially pre-processed by the algorithm using a 2-D median filter (10 by 10 neighborhood) and a subsequent gamma-correction (γ = 0.2) to balance pixel brightness variability across the scale area. By applying the pre-processing operations to the original data shown in Fig. 2a, a smoothed, low spatial frequency content image is generated (Fig. 2b), enabling thus the k-means clustering method to differentiate the scale area from the background. A binary mask delineating the scale area (white color) using the k-means algorithm is presented in Fig. 2c to demonstrate the typical accuracy of the automated image processing approach. A normalized pixel histogram for the pre-processed image is shown explicitly in Fig. 2d, to reveal a high concentration of normalized brightness values between 0.45 and 0.50 corresponding to the background, and an approximately even distribution of pixels in the range of 0.60–0.85 associated predominantly to the scale area. In this case, k-means clustering method has provided the centroid value for each sub-group (i.e., the scale and the background) at 0.718 and 0.472 respectively, as denoted by the dotted red lines in the histogram of Fig. 2d. An average of these centroid values has been estimated at 0.595 (black dotted line), serving as a normalized brightness threshold for the differentiation between the sub-groups. To further investigate the relative performance of the developed k-means based algorithm as regards the accurate selection of the scale area, we have generated a Bland–Altman plot (Fig. 2e) analyzing the agreement between the automated process and a careful manual selection of the scale from recorded CF images. By employing a total number of 40 seabream scales, we have estimated the individual selected area differences between the manual and k-means method and plotted the resulting values as a function of the corresponding mean areas by averaging the results from both approaches. The average difference value between the two methods was found to be equal to 667 pixels (horizontal solid line in Fig. 2e), indicating that the manual selection method results into slightly larger scale area estimations. The 95% confidence intervals for the differences were additionally calculated at − 1728 and 3062 pixels respectively and are indicated by the dashed lines of the Bland–Altmann plot. As the mean area for all scales was equal to 29,148 pixels, we can finally estimate an average of ~ 2.3% difference between the two methods, which demonstrates the excellent agreement of the novel k-means clustering algorithm with traditional manual image processing approaches.

### Investigation of melanin accumulation in different fish species

**Figure 3:**
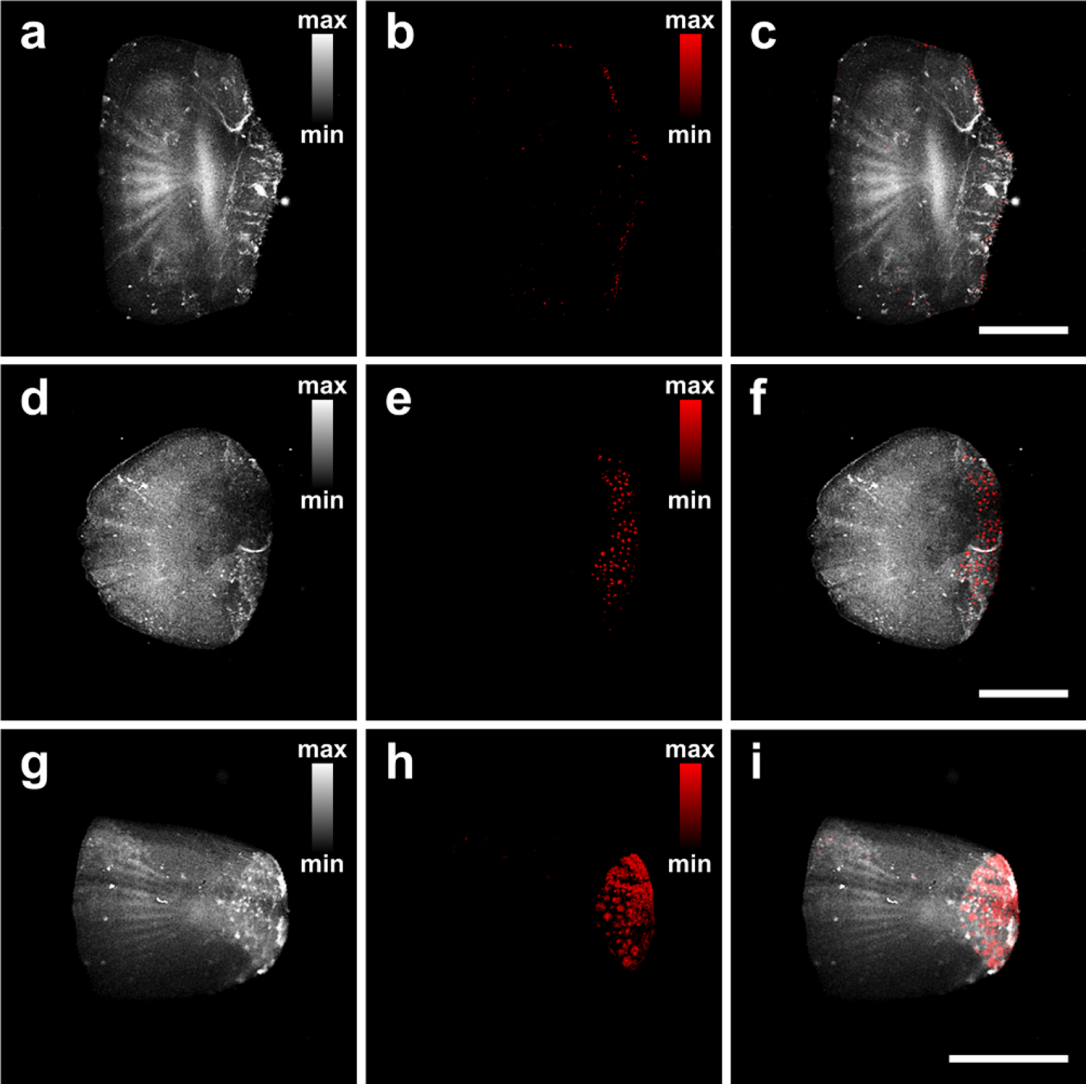
Hybrid label-free imaging of scales belonging to different fish species. (**a**) CF image of a common pandora scale, recording the spatial distribution of emitted AF signals. (**b**) PA image of the same region, indicating a minimal melanin accumulation. (**c**) Hybrid image of the scale incorporating CF (grayscale) and PA (red color) contrast modes. (**d**–**f**) Respective imaging data obtained for a typical gilthead seabream scale. (**g**–**h**) Similar images for a sea bass scale presenting an apparent high melanin accumulation. All scale bars are equal to 2 mm.

To demonstrate the capabilities of the hybrid microscopy system as regards the detection and quantification of melanin accumulation, we have investigated scales that were collected from three different fish species, namely, common pandora, gilthead seabream and European sea bass. A sample of 100 scales per fish species was selected for the statistical processing of melanin’s measurements and the relative comparison of results among the different groups. Figure 3a depicts a CF image of a typical common pandora scale with a field of view equal to 8 by 8 mm^2^, which is sampled using 400 by 400 pixels (pixel size: 20 μm). The scale is generally characterized by a homogeneous AF signal distribution across its extent, demonstrating though, some dispersed small regions of high emission intensity. A PA image of the same region is shown in Fig. 3b, to reveal a small number of scattered melanophores located mainly in the posterior field of the scale. A hybrid contrast image combining CF (grayscale) and PA (red color) modalities is shown in Fig. 3c, providing multiparametric information of the specimen in a label-free diagnostic approach. Similar data are presented in Fig. 3d–f for a gilthead seabream scale using identical imaging parameters. In this case, it can be observed that the PA image (Fig. 3e) demonstrates a larger population of melanophores compared to the common pandora’s scale (Fig. 3b). The individual melanophores (or melanophore aggregates), appear as dispersed bright spots with typical diameters in the order of 100 μm. Finally, hybrid microscopy images are presented for a sea bass scale (Fig. 3g–i), using this time a field of view of 6 by 6 mm^2^ due to the relatively smaller average size of these specimens (pixel size: 15 μm). As it is clear from the PA image (Fig. 3h), the sea bass scale presents a dense accumulation of melanophores in the posterior field, which is significantly higher compared to both of the previous specimens.

**Figure 4:**
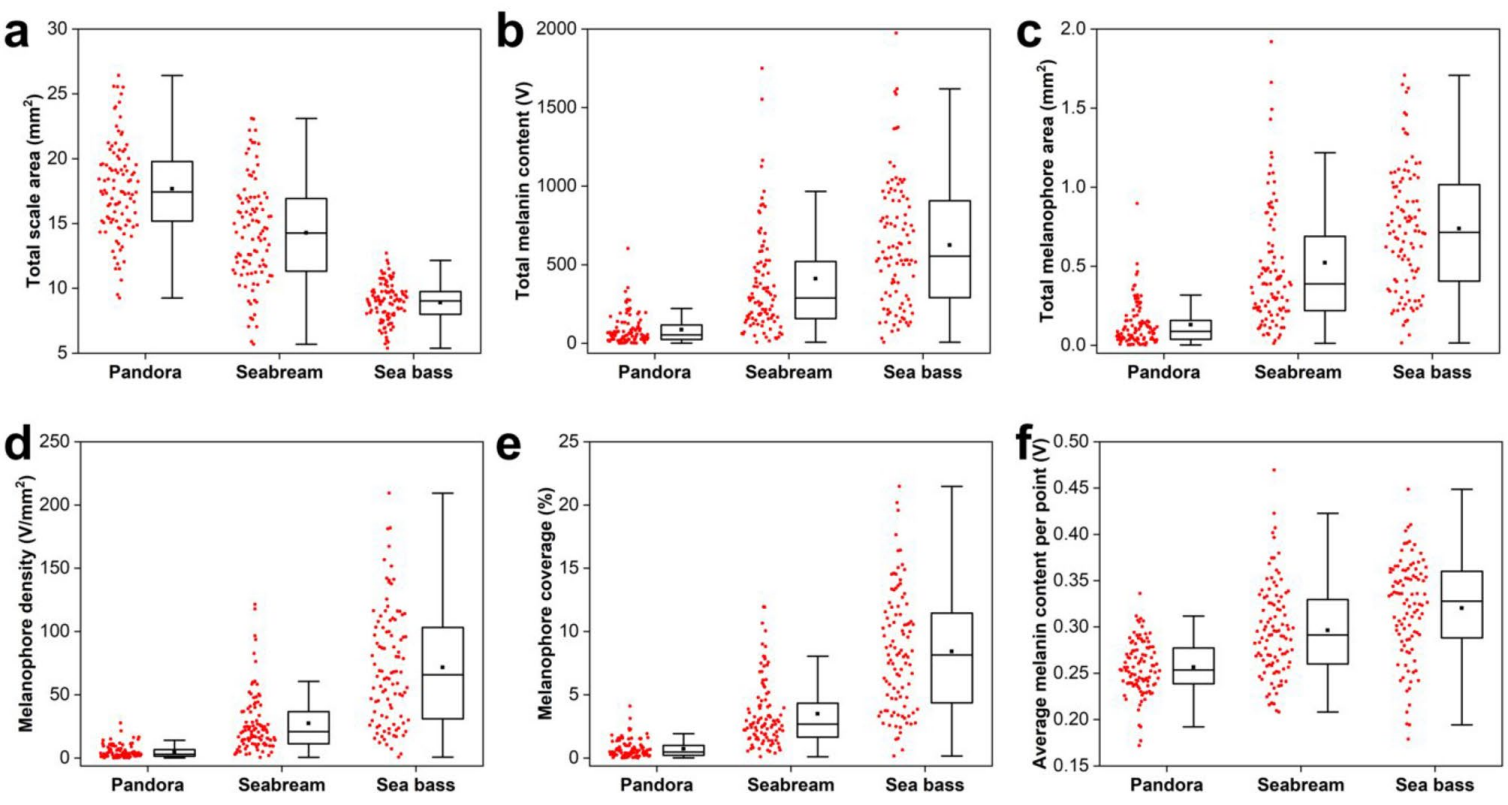
Box plots of melanin accumulation indices for the three investigated fish species (common pandora, gilthead seabream, European sea bass). (**a**) Total scale area in mm^2^. (**b**) Total melanin content in V. (**c**) Total melanophore area in mm^2^. (**d**) Melanophore density in V/mm^2^. (**e**) Percentage of melanophore coverage (%). (**f**) Average melanin content per point (V). A total number of 300 scales (100 per fish category) was measured and processed for the extraction of these results.

Using the developed k-means image processing algorithm and by defining a fixed PA signal threshold for the effective rejection of background noise, we have generated six different indices which aim at the accurate quantification of melanin accumulation in fish scales. These indices include: (a) the total scale area (mm^2^), as extracted directly by the k-means clustering process (see Fig. 2 for more details), (b) the total melanin content (V), which is estimated by summing all PA amplitude contributions exceeding a pre-defined threshold of 0.15 V over the detected scale area, (c) the total melanophore area (mm^2^), given by the summation of pixels with PA values greater than the threshold and the subsequent conversion in mm^2^ using the selected pixel size, (d) the melanophore density (V/mm^2^), derived by taking the ratio between index (b) and (a), (e) the percentage of melanophore coverage (%), calculated similarly by dividing index (c) with (a), and finally (f) the average melanin content per point (V), given by the division of index (b) with the total number of pixels exceeding the threshold in the scale area. The extracted quantified results for the three investigated fish species are graphically presented in the form of boxplots (Fig. 4a–f), where red dots represent the distribution of individual measurements, box corresponds to the interquartile range (IQR; Q3–Q1), horizontal line in the box is the median, black square stands for the mean value, and whiskers, indicating the variability of the statistical sample, extend up to 1.5 * IQR. The estimated mean values and corresponding standard errors for all melanin accumulation indices are explicitly presented in Table 1. To compare the estimated means for the three fish categories, we have performed one-way ANOVA with respective post-hoc tests (Bonferroni and Tukey), confirming that resulting differences are statistically significant in all indices (*p* < 0.001). Common pandora group demonstrated the lowest melanin accumulation levels, whereas sea bass category revealed the highest ones, both in terms of absolute content and relative density, as expressed by all extracted quantitative measures (Fig. 4b–e).

**Table 1:** Mean values of melanin accumulation indices for the investigated fish groups.

Fish group	Scale area (mm^2^)	Melanin content (V)	Melanophore area (mm^2^)	Melanophore density (V/mm^2^)	Coverage (%)	Melanin content/point (V)
Common pandora	17.67 (± 0.36)	86.83 (± 9.63)	0.129 (± 0.014)	4.83 (± 0.50)	0.718 (± 0.071)	0.256 (± 0.003)
Seabream	14.28 (± 0.40)	411.48 (± 42.50)	0.521 (± 0.047)	27.32 (± 2.38)	3.486 (± 0.262)	0.296 (± 0.005)
Sea bass	8.91 (± 0.15)	624.36 (± 39.86)	0.737 (± 0.042)	71.60 (± 4.57)	8.419 (± 0.468)	0.320 (± 0.005)

Value in parenthesis represents the standard error of the mean. All differences are statistically significant at 0.001 level.

As a next step, we evaluated the performance of the trained machine learning model on the test set (unseen data during training) as described in the respective section of “Materials and methods” section. Due to the relatively limited amount of data, we need to make sure that the performance of our model does not depend on the preparation of the training validation and test sets. Thus, we further examine the robustness of the predictive capabilities of the trained model by applying a cross validation (CV) method. For this reason, we used 100 different, randomly assigned, training, validation and test sets of 216, 24 and 60 images respectively in each set, to train and evaluate our model. The average and the standard deviation of the precision, recall, F1 and accuracy over 100 different sets is shown in Table 2.

**Table 2:** The average and the standard deviation, in parenthesis, of the precision, recall, f1 and accuracy over 100 different sets for training, validation and test.

	Common pandora	Sea bream	Sea bass
Precision	0.89 (± 0.08)	0.80 (± 0.10)	0.87 (± 0.08)
Recall	0.92 (± 0.07)	0.75 (± 0.10)	0.85 (± 0.10)
F1	0.90 (± 0.05)	0.76 (± 0.09)	0.85 (± 0.07)
Accuracy	0.85 (± 0.05)		

The average accuracy of the model is 85%, with a standard deviation of 5%, revealing the stability of the performance of the model. The model achieves high scores in the classification of the common pandora and the sea bass classes and lower scores in the gilthead seabream one. This is because the characteristics of melanin accumulation in seabreams are in between of those of the common pandora’s and sea bass’ ones as shown in Fig. 4, and thus it is harder for the model to correctly classify the gilthead seabream class. Nevertheless, in all cases, the deviation of the average scores is at most 10%, indicating the generalizability of the proposed model.

### Investigation of melanin in porgies

**Figure 5:**
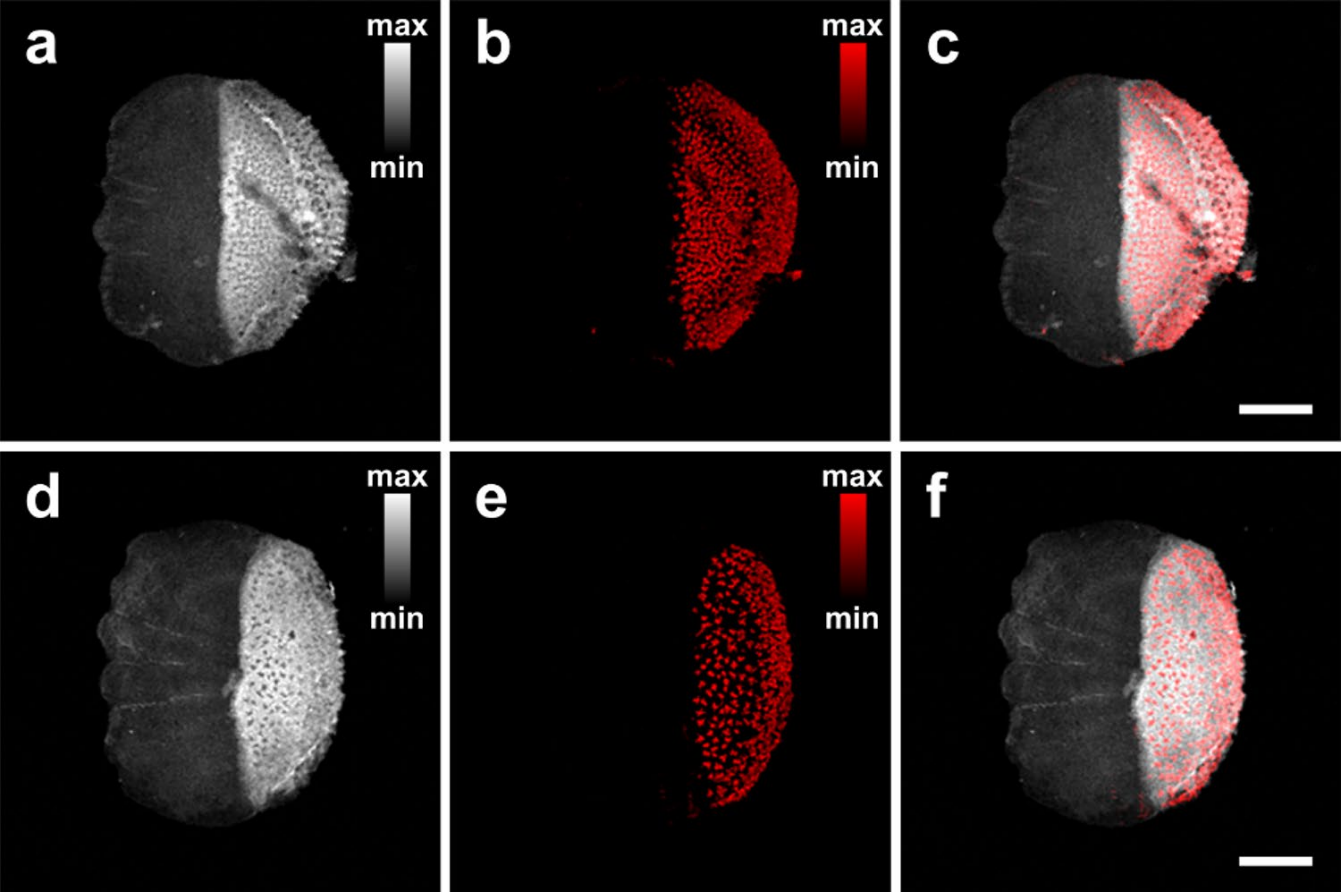
Scales of porgies from different fish suppliers. (**a**) CF image of a scale extracted from a fish of S1 group. (**b**) Corresponding PA image depicting a dense distribution of melanin. (**c**) Hybrid reconstruction merging CF (grayscale) and PA (red color) images. (**d**) CF image of a scale extracted from S2 group. (**e**) PA image revealing a sparser distribution of melanophores. (**f**) Respective hybrid image of the sample. Both scale bars are equal to 1 mm.

Having verified the significant melanin accumulation variability among different fish species by means of the hybrid microscope, we proceeded to the investigation of scales extracted from porgies purchased from different fish suppliers in Crete (S1 and S2). A total number of 40 scales (20 per group) were imaged by means of the hybrid microscope to extract the respective melanin accumulation indices, similarly to the previous study involving different fish species. In Fig. 5a, we present a CF image (8 by 8 mm^2^; pixel size: 20 μm) of a porgy scale belonging to the S1 group, to observe an intense emission of AF in the posterior field, as a result of the increased collagen concentration. The strong AF region is additionally characterized by a large number of dispersed spots demonstrating lower emissions than the average signal background. Moreover, Fig. 5b depicts an extended and dense accumulation of melanophores through the recording of high-amplitude PA signals, whose spatial distribution virtually coincides with the previously detected low AF spots. In this manner, Fig. 5b provides complementary contrast in respect to the CF image, as can been validated by the hybrid reconstruction of the scale presented in Fig. 5c. A CF image of a typical scale belonging to the S2 group is shown in Fig. 5d, to reveal a highly similar AF emission behavior to the previous scale (Fig. 5a). Nevertheless, the respective PA image (Fig. 5e) indicates the presence of melanophores at a substantially lower spatial density in comparison to the previously mapped distribution (Fig. 5b). The hybrid reconstruction incorporating the two contrast modes is finally shown in Fig. 5f, to delineate scale’s morphological and optical absorption information with high resolution and imaging specificity.

**Figure 6:**
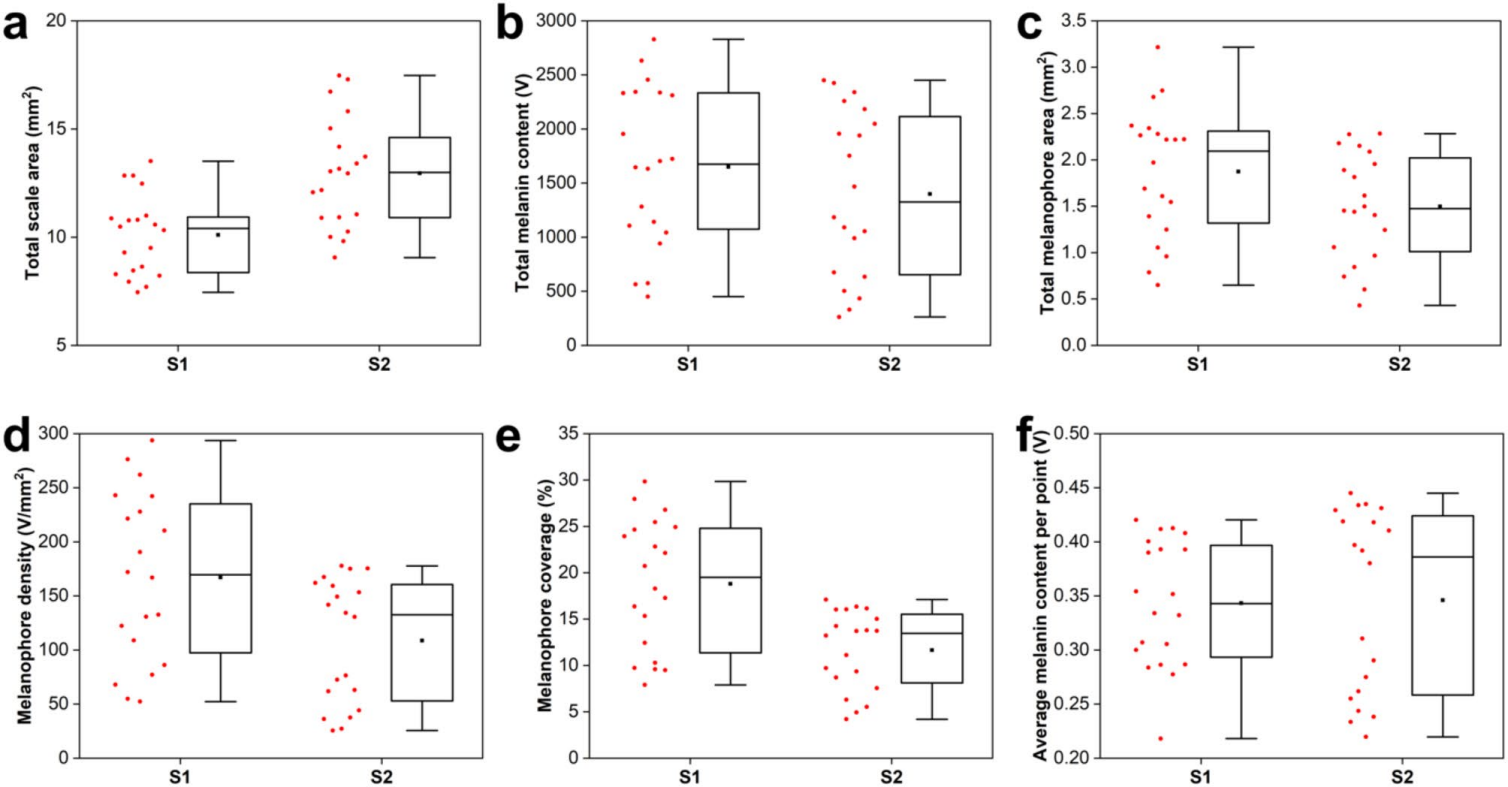
Box plots of melanin accumulation indices for porgies purchased from different fish suppliers. (**a**) Total scale area in mm^2^. (**b**) Total melanin content in V. (**c**) Total melanophore area in mm^2^. (**d**) Melanophore density in V/mm^2^. (**e**) Percentage of melanophore coverage (%). (**f**) Average melanin content per point (V). A total number of 40 scales (20 per group) was measured and processed for the extraction of these results.

The k-means automated signal quantification algorithm was employed for the estimation of the mean melanin accumulation indices in the two fish groups (S1 and S2). T-tests were additionally conducted to investigate the statistical significance of the differences between the groups. The results of the processing are presented both graphically as box plots (Fig. 6a–f) and in analytical form (Table 3), to reveal statistically significant differences between the means of the groups as regards the total scale area (*p* < 0.001), the melanophore density (*p* < 0.05) and coverage (*p* < 0.001).

**Table 3:** Mean values of melanin accumulation indices for the investigated fish groups.

Fish group	Scale area (mm^2^)**	Melanin content (V)	Melanophore area (mm^2^)	Melanophore density (V/mm^2^)*	Coverage (%)**	Melanin content/point (V)
S1	10.10 (± 0.41)	1650.12 (± 164.60)	1.873 (± 0.157)	166.98 (± 17.45)	18.797 (± 1.580)	0.343 (± 0.013)
S2	12.95 (± 0.57)	1398.65 (± 172.12)	1.497 (± 0.130)	108.53 (± 12.88)	11.639 (± 0.954)	0.346 (± 0.019)

Value in parenthesis represents the standard error of the mean.

*Statistically significant at 0.05 level.

**Statistically significant at 0.001 level.

## Discussion and conclusions

The present study introduced a novel hybrid CF and optical-resolution PA microscopy methodology to quantify melanophore number and melanin content in fish scales. Using this approach, it was possible to quantify inter-specific differences in melanophore number and melanin content in scales from three different marine teleost fish species. Moreover, images produced using this methodology were automatically classified to the respective species with high accuracy when analyzed using a machine learning model for image classification.

Among skin pigments, melanin is present in almost all vertebrates, apart from albino forms, and affects the lightness of the skin coloration. Fish display a huge color diversity, with skin coloration being crucial for their biology, including health^12,13,63,64^, conspecific recognition and social behavior^65^, shoaling preference^66^, and mate choice^67,68^. However, the currently available techniques to quantify melanin concentration and/or measure the number and coverage of melanophores in fish skin and scales are laborious and not automated. Apart from that, in order to measure melanin content with traditional methods fish have to be sacrificed to collect skin samples. The method developed and evaluated in the present study provides an accurate, repeatable and fast way to measure both melanin concentration and melanophore number and area coverage in fish scales, while additionally being non-invasive since it can be performed in single scales without the need to sacrifice fish.

When this imaging technique was coupled with scale photographs obtained using an optical stereoscope the signal of melanin, and therefore the melanosomes, in the PA images was scattered in the same way as in the optical image. The accompanying development of an automated image processing algorithm based on standard k-means clustering method allowed for a fast and reliable estimation of the fish scale area and the melanin and melanophore concentrations.

In order to examine the capability of this technique to quantify inter-specific differences, scales from three marine teleost showing different skin lightness in their dorsal area were analyzed. These species were the European sea bass (dark dorsal area), the common pandora (light dorsal area) and the gilthead seabream (intermediate lightness in dorsal area). The PA imaging not only recorded differences in the number of melanophores between the three species, as was expected, but also managed to quantify the total melanin content of each scale, which also differed between the three species. On top of that, when the images produced by the PA system were analyzed using a machine-learning algorithm, it was possible to distinguish the species to which each scale belong to with high accuracy.

Due to the fact that melanin is involved in many processes, not only restricted to skin coloration and its derivatives, but also to metal ion homeostasis and antioxidant function^69^, response to parasites and pathogens^64,70^ and can be used as bio-detectors in pharmaceutical and pollutants research^69,71^, its measurement is gaining much attention in fish and therefore this novel quantification technique can find many applications. A few examples include, but are not restricted to (1) scale culture assays testing the effects of substances in melanophores, as in^71^, (2) the effects of parasitic load on the demelanization of fish, as in^64,70^ and (3) the effects of climate change on population dynamics, as in^16^. With climate change leading to increased UV-radiation, due to the thin ozone layer, and water temperatures, due to global warming, fishes might suffer color changes, such as hypermelanosis, or move to deeper waters in order to cope with the environmental conditions formed. Natural selection of darker colors might be the case in the future and the need to monitor and study those changes will lead to an increased need to measure skin color and melanin contents. Moreover, this technique could find application in aquaculture, since skin coloration affects the economic value of harvested fish. Red porgy, for instance, is a species characterized by its light red coloration in the wild, but a significant darkening when cultured^2,20^. Studies in red porgy (*Pagrus pagrus*) have shown that it requires low light intensity rearing conditions to avoid hypermelanosis^2^. In addition, when reared in 45 m submerged sea cages, red porgy presented significantly brighter skin color and lower skin melanin content compared with the control group reared in a surface cage^72^.

Such differences in biological, for instance health and the depth that fish dwell, as well as managerial, as for example the site of fishing in wild-caught or the site of culture in cultured fish, factors can potentially affect the skin color and melanin content of fish sold in the market under the same common species name. Indeed, in this study it was shown that the presented methodology was able to detect differences in porgies purchased from different suppliers in terms of their melanophore density and coverage (%). Moreover, the developed machine-learning algorithm was able to automatically attribute the scales to the correct group with high accuracy. It is, in this regard, shown that this methodology is highly precise in its analysis and managed to attribute small changes in the layout of fish scales in terms of their melanin content and melanophore coverage.

The technological capabilities of the demonstrated imaging system could be further upgraded using tunable nanosecond laser sources for the efficient PA excitation of several chromatophores in various types of fish scales, presenting strong optical absorption in different spectral regions. The multi-wavelength PA excitation may be enhanced with custom-developed spectral unmixing algorithms for the identification and relative concentration measurement of the investigated pigments. In addition, the generation of a calibration curve between the recorded PA amplitude and the local molar concentration of melanin could be performed in a future study, providing thus absolute values of the total melanin content in fish scales. Furthermore, an upgraded version of the presented hybrid microscope could be employed for the direct imaging of scales in an anesthetized fish in vivo, eliminating the need of sampling. To achieve this, the PA microscopy technique has to be re-designed in reflection mode, either by using a combination of prisms or a reflective objective, in order to couple both excitation light and ultrasonic detection on the same side. Finally, the mechanical raster scanning procedures can be replaced by galvanometric mirror beam scanning, allowing for the rapid collection of large datasets, which in turn may result in the improved training of the employed CNN algorithms, increasing thus the accuracy of scale classification. Such further developments, combined with the already demonstrated performance of the developed hybrid label-free microscope, are expected to expand substantially the applicability of the presented technique as regards the accurate quantification of chromatophores in various fish species.

## Data Availability

The datasets generated and analyzed during the current study are available from the corresponding author on reasonable request.

## References

[1] Fujii R (2000). The regulation of motile activity in fish chromatophores. Pigment Cell Res..

[2] Pavlidis M, Papandroulakis N, Divanach P (2006). A method for the comparison of chromaticity parameters in fish skin: Preliminary results for coloration pattern of red skin Sparidae. Aquaculture.

[3] Sugimoto M (2002). Morphological color changes in fish: Regulation of pigment cell density and morphology. Microsc. Res. Tech..

[4] Pavlidis M, Karkana M, Fanouraki E, Papandroulakis N (2008). Environmental control of skin colour in the red porgy, *Pagrus pagrus*. Aquac. Res..

[5] Szisch V, van der Salm AL, Wendelaar Bonga SE, Pavlidis M (2002). Physiological colour changes in the red porgy, *Pagrus pagrus*, following adaptation to blue lighting spectrum. Fish Physiol. Biochem..

[6] Vissio PG, Darias MJ, Di Yorio MP, Pérez Sirkin DI, Delgadin TH (2021). Fish skin pigmentation in aquaculture: The influence of rearing conditions and its neuroendocrine regulation. Gen. Comp. Endocrinol..

[7] Johansen R, Needham JR, Colquhoun DJ, Poppe TT, Smith AJ (2006). Guidelines for health and welfare monitoring of fish used in research. Lab. Anim..

[8] Pavlidis MA, Chatzifotis S, Adachi K, Pavlidis M, Mylonas C (2011). Pigmentation physiology and discoloration problems. Sparidae Biology and Aquaculture of Gilthead Sea Bream other Species.

[9] Colihueque N, Araneda C (2014). Appearance traits in fish farming: Progress from classical genetics to genomics, providing insight into current and potential genetic improvement. Front. Genet..

[10] Liao Q (2017). Monitoring red sea bream scale fluorescence as a freshness indicator. Fishes.

[11] Papaharisis L, Tsironi T, Dimitroglou A, Taoukis P, Pavlidis M (2019). Stress assessment, quality indicators and shelf life of three aquaculture important marine fish, in relation to harvest practices, water temperature and slaughter method. Aquac. Res..

[12] Kittilsen S, Johansen IB, Braastad BO, Øverli Ø (2012). Pigments, parasites and personalitiy: Towards a unifying role for steroid hormones?. PLoS ONE.

[13] Côte J (2018). Melanin-based coloration and host–parasite interactions under global change. Proc. R. Soc. B Biol. Sci..

[14] Khan UW (2016). A novel role for pigment genes in the stress response in rainbow trout (*Oncorhynchus mykiss*). Sci. Rep..

[15] Rodríguez FH (2019). Genetic (co)variation in skin pigmentation patterns and growth in rainbow trout. Animal.

[16] Roulin A (2014). Melanin-based colour polymorphism responding to climate change. Glob. Change Biol..

[17] Pulcini D, Capoccioni F, Franceschini S, Martinoli M, Tibaldi E (2020). Skin pigmentation in gilthead seabream (*Sparus aurata* L.) fed conventional and novel protein sources in diets deprived of fish meal. Animals.

[18] Wilson JF, Dodd JM (1973). The role of melanophore-simulating hormone in melanogenesis in the dogfish, *Scyliorhinus canicula* L. J. Endocrinol..

[19] Sugimoto M (1993). Morphological color changes in the medaka, *Oryzias latipes*, after prolonged background adaptation—I. Changes in the population and morphology of melanophores. Comp. Biochem. Physiol. A Mol. Integr. Physiol..

[20] Chatzifotis S (2005). The effect of different carotenoid sources on skin coloration of cultured red porgy (*Pagrus pagrus*). Aquac. Res..

[21] Ito S, Wakamatsu K (2003). Quantitative analysis of eumelanin and pheomelanin in humans, mice, and other animals: A comparative review. Pigment Cell Res..

[22] Adachi K, Kato K, Ito S, Ishimaru K (2005). The histological analysis, colorimetric evaluation, and chemical quantification of melanin content in ‘suntanned’ fish. Pigment Cell Res..

[23] Chatzifotis S, vaz Juan I, Kyriazi P, Divanach P, Pavlidis M (2011). Dietary carotenoids and ksin melanin content influence the coloration of farmed red porgy (*Pagrus pagrus*). Aquac. Nutr..

[24] Ntziachristos V (2010). Going deeper than microscopy: The optical imaging frontier in biology. Nat. Methods.

[25] Yao J, Wang LV (2014). Sensitivity of photoacoustic microscopy. Photoacoustics.

[26] Yao J, Wang LV (2013). Photoacoustic microscopy. Laser Photonics Rev..

[27] Jeon S, Kim J, Lee D, Baik JW, Kim C (2019). Review on practical photoacoustic microscopy. Photoacoustics.

[28] Chen J (2020). Confocal visible/NIR photoacoustic microscopy of tumors with structural, functional, and nanoprobe contrasts. Photonics Res..

[29] Dai Y (2020). Metastatic status of sentinel lymph nodes in breast cancer determined with photoacoustic microscopy via dual-targeting nanoparticles. Light Sci. Appl..

[30] Xia J, Kim C, Lovell JF (2015). Opportunities for photoacoustic-guided drug delivery. Curr. Drug Targets.

[31] Zhang HF, Puliafito CA, Jiao S (2011). Photoacoustic ophthalmoscopy for in vivo retinal imaging: current status and prospects. Ophthalmic Surg Lasers Imaging.

[32] Nguyen VP, Paulus YM (2018). Photoacoustic ophthalmoscopy: Principle, application, and future directions. J. Imaging.

[33] Wang LV, Wu H-I (2007). Biomedical Optics: Principles and Imaging.

[34] Wissmeyer G, Pleitez MA, Rosenthal A, Ntziachristos V (2018). Looking at sound: Optoacoustics with all-optical ultrasound detection. Light Sci. Appl..

[35] Xia J, Yao J, Wang LV (2014). Photoacoustic tomography: Principles and advances. Prog. Electromagn. Res..

[36] Paproski RJ, Heinmiller A, Wachowicz K, Zemp RJ (2014). Multi-wavelength photoacoustic imaging of inducible tyrosinase reporter gene expression in xenograft tumors. Sci. Rep..

[37] Xu D, Yang S, Wang Y, Gu Y, Xing D (2016). Noninvasive and high-resolving photoacoustic dermoscopy of human skin. Biomed. Opt. Express.

[38] Liu X (2015). Optical coherence photoacoustic microscopy for in vivo multimodal retinal imaging. Opt. Lett..

[39] He Y (2016). In vivo label-free photoacoustic flow cytography and on-the-spot laser killing of single circulating melanoma cells. Sci. Rep..

[40] Deán-Ben XL, Razansky D (2021). Optoacoustic imaging of the skin. Exp. Dermatol..

[41] Kneipp M (2015). Volumetric tracking of migratory melanophores during zebrafish development by optoacoustic microscopy. Mech. Dev..

[42] Li L (2017). Single-impulse panoramic photoacoustic computed tomography of small-animal whole-body dynamics at high spatiotemporal resolution. Nat. Biomed. Eng..

[43] Lister T, Wright P, Chappell P (2010). Spectrophotometers for the clinical assessment of port-wine stain skin lesions: A review. Lasers Med. Sci..

[44] Dasa MK (2018). High-pulse energy supercontinuum laser for high-resolution spectroscopic photoacoustic imaging of lipids in the 1650–1850 nm region. Biomed. Opt. Express.

[45] Sekar SKV (2017). Diffuse optical characterization of collagen absorption from 500 to 1700 nm. J. Biomed. Opt..

[46] Bergmann F, Foschum F, Marzel L, Kienle A (2021). Ex vivo determination of broadband absorption and effective scattering coefficients of porcine tissue. Photonics.

[47] Tserevelakis GJ, Tsagkaraki M, Zacharakis G (2016). Hybrid photoacoustic and optical imaging of pigments in vegetative tissues. J. Microsc..

[48] Tserevelakis GJ, Avtzi S, Tsilimbaris MK, Zacharakis G (2017). Delineating the anatomy of the ciliary body using hybrid optical and photoacoustic imaging. J. Biomed. Opt..

[49] Tserevelakis GJ (2020). Hybrid autofluorescence and photoacoustic label-free microscopy for the investigation and identification of malignancies in ocular biopsies. Opt. Lett..

[50] Tserevelakis GJ, Soliman D, Omar M, Ntziachristos V (2014). Hybrid multiphoton and optoacoustic microscope. Opt. Lett..

[51] Soliman D, Tserevelakis GJ, Omar M, Ntziachristos V (2015). Combining microscopy with mesoscopy using optical and optoacoustic label-free modes. Sci. Rep..

[52] Rao B, Soto F, Kerschensteiner D, Wang LV (2014). Integrated photoacoustic, confocal, and two-photon microscope. J. Biomed. Opt..

[53] Hosseinaee Z, Tummon Simmons JA, Reza PH (2021). Dual-modal photoacoustic imaging and optical coherence tomography [review]. Front. Phys..

[54] Dadkhah A, Zhou J, Yeasmin N, Jiao S (2019). Integrated multimodal photoacoustic microscopy with OCT-guided dynamic focusing. Biomed. Opt. Express.

[55] Zhang W (2020). Simultaneous photoacoustic microscopy, spectral-domain optical coherence tomography, and fluorescein microscopy multi-modality retinal imaging. Photoacoustics.

[56] Park J (2021). Quadruple ultrasound, photoacoustic, optical coherence, and fluorescence fusion imaging with a transparent ultrasound transducer. Proc. Natl. Acad. Sci. U.S.A..

[57] Kaleta K (2009). Morphological analysis of chromatophores in the skin of trout. Bull. Vet. Inst. Pulawy.

[58] Szydlowski P, Madej JP, Mazurkiewicz-Kania M (2017). Histology and ultrastructure of the integumental chromatophores in tokay gecko (*Gekko gecko*) (Linnaeus, 1758) skin. Zoomorphology.

[59] Arvai, K. K-Means Clustering in Python: A Practical Guide. *Real Python*. https://realpython.com/k-means-clustering-python/ (2020).

[60] Chollet, F. et al., *Keras*. https://keras.io/ (2015).

[61] Abadi, M. et al. Tensorflow: A system for large- scale machine learning, *12th {USENIX} Symposium on Operating Systems Design and Implementation ({OSDI} 16)*, **265** (2016).

[62] Kingma, D.P. & Ba, J. Adam: A method for stochastic optimization. https://arxiv.org/abs/1412.6980 (2017).

[63] Milinski M, Bakker TCM (1990). Female sticklebacks use male coloration in mate choice and hence avoid parasitized males. Nature.

[64] Ness JH, Foster SA (1999). Parasite-associated phenotype modifications in threespine stickleback. Oikos.

[65] Grosenick L, Clement T, Fernald R (2007). Fish can infer social rank by observation alone. Nature.

[66] McRobert SP, Bradner J (1998). The influence of body coloration on shoaling preferences in fish. Anim. Behav..

[67] Kodric-Brown A (1998). Sexual dichromatism and temporary color changes in the reproduction of fishes. Am. Zool..

[68] Seehausen O, Mayhew PJ, van Alphen JJM (1999). Evolution of colour patterns in East African cichlid fish. J. Evol. Biol..

[69] Nilsson Sköld H, Aspengren S, Wallin M (2013). Rapid color change in fish and amphibians—function, regulation, and emerging applications. Pigment Cell Melanoma Res..

[70] Pawluk RJ, De Leaniz CG, Cable J, Tiddeman B, Consuegra S (2019). Colour plasticity in response to social context and parasitic infection in a self-fertilizing fish. R. Soc. Open Sci..

[71] Lennquist A, Mårtensson Lindblad LGE, Hedberg D, Kristiansson E, Förlin L (2010). Colour and melanophore function in rainbow trout after long term exposure to the new antifoulant medetomidine. Chemosphere.

[72] Papandroulakis N (2012). Installation, operation and evaluation of a submerged cage at 45M depth in Crete for the rearing of red porgy *Pagrus pagrus*. Aquac. Res..

